# Forced differentiation *in vitro* leads to stress-induced activation of DNA damage response in hiPSC-derived chondrocyte-like cells

**DOI:** 10.1371/journal.pone.0198079

**Published:** 2018-06-04

**Authors:** Ewelina Stelcer, Katarzyna Kulcenty, Marcin Rucinski, Karol Jopek, Magdalena Richter, Tomasz Trzeciak, Wiktoria Maria Suchorska

**Affiliations:** 1 Radiobiology Laboratory, Greater Poland Cancer Centre, Poznan, Poland; 2 The Postgraduate School of Molecular Medicine, Medical University of Warsaw, Warsaw, Poland; 3 Department of Electroradiology, Poznan University of Medical Sciences, Poznan, Poland; 4 Department of Histology and Embryology, Poznan University of Medical Sciences, Poznan, Poland; 5 Department of Orthopedics and Traumatology, Poznan University of Medical Sciences, Poznan, Poland; University of Texas at Austin Dell Medical School, UNITED STATES

## Abstract

A human induced pluripotent stem cell line (GPCCi001-A) created by our group was differentiated towards chondrocyte-like cells (ChiPS) via monolayer culturing with growth factors. ChiPS are promising because they have the potential to be used in tissue engineering to regenerate articular cartilage. However, their safety must be confirmed before they can be routinely used in regenerative medicine. Using microarray analysis, we compared the ChiPS to both GPCCi001-A cells and chondrocytes. The analysis showed that, compared to both GPCCi001-A cells and chondrocytes, the expression of genes engaged in DNA damage and in the tumor protein p53 signalling pathways was significantly higher in the ChiPS. The significant amount of DNA double strand breaks and increased DNA damage response may lead to incomplete DNA repair and the accumulation of mutations and, ultimately, to genetic instability. These findings provide evidence indicating that the differentiation process *in vitro* places stress on human induced pluripotent stem cells (hiPSCs). The results of this study raise doubts about the use of stem cell-derived components given the negative effects of the differentiation process *in vitro* on hiPSCs.

## Introduction

Stem cells (SCs), particularly human induced pluripotent SCs (hiPSCs), constitute a real hope to better understand the pathogenesis and improve the treatment of many disorders (e.g. neurodegenerative, neurovascular, and cardias diseases) that are unresponsive to current treatments [1]. HiPSCs hold great potential in regenerative medicine due to their potentially unlimited self-renewal capacity and ability to give rise to all of the somatic lineages in the body [2]. HiPSCs can be cultured in two main ways: feeder-dependent and feeder-free systems. In the feeder-dependent system, the cells are placed on a layer of inactivated murine embryonic fibroblasts as feeder cells in a medium supplemented with fetal bovine serum or proprietary replacements such as KnockOut Serum Replacement [3]. However, this approach presents a risk of contamination with animal pathogens and is thus considered unsuitable for clinical applications. By contrast, feeder-free culture systems represent a significant improvement over feeder-dependent systems, making these cells and their derivatives more suitable for use in clinical practice [4]. However, it is essential that Good Manufacturing Practices (GMP)—which provide strict conditions for production of these cells—be followed when generating hiPSCs in a feeder-free culture[5]. GMP validation—which involves genetic stability analyses, virus and pathogen testing and method of derivation—is crucial before hiPSCs-based cell therapies can move from bench to bedside. This extensive characterization and validation process helps to ensure patient safety. Conceivably, for some patients, bioproducts based on hiPSCs may offer a viable treatment in the future [6].

HiPSCs can be differentiated into specific lineages—cardiac, osteogenic, chondrogenic, and neural—that possess distinct features and characteristics. The differentiation process *in vitro* can be achieved with either two- or three-dimensional (3D) cell culture methods [7,8]. In 3D cultures, such as differentiation using scaffolds or bioreactors, cell-cell contacts and cell-matrix interactions mimicking the native environment play an important role [9]. For this reason, 3D cultures may be preferred because they improve and enhance the effectiveness of differentiation protocols [10]. The most common methods of hiPSC differentiation *in vitro* are embryoid body formation [11] and micromass [12] and pellet culture [13]. In these methods, hiPSCs are differentiated in the medium with the addition of exogenous growth factors (GFs). The most common GFs are epidermal growth factor (EGF), fibroblast growth factor (FGF), bone morphogenetic protein 4 (BMP-4), transforming growth factor 3 (TGF-β3), and Activin A [14].

According to the published data, differentiation *in vitro* leads to the activation of the tumor protein p53 signalling pathway that controls the course of the differentiation process *in vitro* throughout the inter alia downregulation of agents responsible for pluripotency (e.g., Nanog). The differentiation process is also accompanied by telomere shortening, an increase in the levels of reactive oxygen species (ROS) and deficient DNA repair activities [15,16]. However, there is notable lack of data on DNA damage and p53-associated signalling pathways in hiPSCs during chondrogenic differentiation. In this context, we conducted the present study. The main aim was to evaluate how the chondrogenic differentiation process influences essential genes engaged in DNA damage and p53 signalling pathways and to compare these aspects to both mature chondrocytes and hiPSCs created by our group. We performed a global gene expression analysis using the Affymetrix platform in order to perform this comparison.

Recently, our group generated a hiPS cell line (GPCCi001-A) from primary human dermal fibroblasts using a lentiviral system [17]; these cells were subsequently differentiated towards chondrocyte-like cells (ChiPS) [18]. Our protocol involves a 3-week monolayer culture with supplementation with a relatively small amount of pro-mesodermal (e.g., BMP-4) and chondrogenic (e.g., TGF-β3) GFs. The advantage of this approach over other protocols is that it does not require any additional steps (such as formation of embryoid bodies) and is thus a time- and cost-effective approach.

The present work provides a comprehensive understanding of the processes involved in the formation of DNA damage and stimulation of the p53 signalling pathway, which is activated during chondrogenesis *in vitro* of hiPSCs. Our study confirms that hiPSCs present decreased DNA damage-induced stress mechanisms, which are characteristics of pluripotent stem cells whose capacity to maintain a mutation-free cell population is high. By contrast, hiPSC-derived chondrocytes (ChiPS) are characterized by increased expression of genes involved in DNA damage and p53 mechanisms. These findings raise important questions concerning the genetic stability of cells derived from hiPSCs (including ChiPS), which show a notable downregulation of stress defense mechanisms after undergoing chondrogenic differentiation.

The global gene expression profile of ChiPS were compared to the hiPSCs (GPCCi001-A cell line) and to two cell lines of mature chondrocytes: HC-402-05a (Merck Millipore, Germany) (HC) and primary articular cartilage chondrocytes (ACC).

## Materials and methods

### Ethics

Primary articular cartilage chondrocytes (ACC) used in this study were obtained from Prof. Kruczynski’s at the Department Orthopedics and Traumatology, Poznan University of Medical Sciences. Department of Orthopedics and Traumatology obtained an informed consent from tissue donors.

### Culture of hiPSCs

The hiPSCs were seeded on 10 cm Petri dishes in Matrigel (BD Biosciences, Franklin Lakes, NJ, USA) that had previously been coated with inactivated murine embryonic fibroblasts as a feeder layer (1×10^6^). Following 24 h preparation of the feeder layer, hiPSCs were seeded at 2×10^6^ in hiPSC growth medium: Dulbecco's modified Eagle's medium (DMEM) F12 with L-glutamine (Merck Millipore, Darmstadt, Germany), 20% knockout serum replacement (Thermo Fisher Scientific, Inc., Waltham, MA, USA), 1% non-essential amino acids (Merck Millipore), 0.1 mM β-mercaptoethanol (Merck Millipore), 1% penicillin/streptomycin (P/S; Merck Millipore). Prior to use, the medium was supplemented with fibroblast growth factor 2 (FGF-2; 10 ng/ml; Merck Millipore).

### Culture of chondrocytes

The primary chondrocytes and HC-402-05 cell line were cultured in 0.1% gelatin (Merck Millipore) in DMEM F12 with L-glutamine (Merck Millipore), 10% FBS (Biowest), and 1% P/S (Merck Millipore).

### Chondrogenic differentiation of hiPSCs

GPCCi001-A cells were subjected to chondrogenic differentiation *in vitro* according to a previously established protocol [18]. To obtain chondrocyte-like cells, the hiPSCs were stimulated in a mesodermal/chondrogenic medium supplemented with selected GFs in a monolayer culture for 21 days. The following GFs were used: PDGF-BB (10 ng/ml) (PeproTech, UK); FGF-2 (10 ng/ml); BMP-4 (10 ng/ml) (ImmunoTools, Germany); TGF-β3 (10 ng/ml) (ImmunoTools, Germany); and IGF-1 (10 ng/ml) (Peprotech, UK). The generated ChiPS cells were analyzed at the mRNA and protein levels to confirm their chondrogenic potential. Next, the whole transcriptome of these cells was obtained using the Affymetrix platform.

### Immunofluorescence analysis

The cells were transferred into a 0.1% gelatin-coated 48-well plate for 48 h and then washed with phosphate buffered saline (PBS) (Sigma Aldrich, MO, USA) and fixed for 20 min in 4% formaldehyde (CHEMPUR, POLAND) (400 μl of formaldehyde per well). Then, the cells were rinsed with PBS containing 1% bovine serum albumin (BSA) (Sigma Aldrich, MO, USA) and incubated for 30 min in PBS containing 1% BSA and 0.2% Triton X-100 (Sigma Aldrich, MO, USA). After 30 min, the cells were washed with PBS containing 1% BSA. The primary antibodies were diluted in PBS containing 1% BSA and 0.2% Triton X-100 and the cells were incubated overnight at 4°C with the following primary antibodies (all from Abcam PLC, U.K.): type II collagen (1:100) (ab34712); SSEA4 (1:100) (ab16287). After conjugation with the primary antibodies, the cells were rinsed three times with PBS containing 1% BSA. The following secondary antibody was diluted with 1% BSA in PBS and incubated in the dark for 1 h at 37°C: rabbit polyclonal (1:500) (711-546-152) and mouse monoclonal antibodies (1:500) (715-545-150) (Jackson ImmunoResearch, PA, USA). After the cells were washed three times with 1% BSA in PBS, they were stained for 5 min with diamidino-2-phenylindole dye (DAPI) (Sigma Aldrich, MO, USA) solution in water (1:10000) and then washed with PBS before undergoing microscope analysis.

### RNA extraction

Total RNA was isolated according to the Chomczynski method using TRI Reagent (Sigma, St Louis, MO, USA) and the RNeasy Mini Elute cleanup Kit (Qiagen, Hilden, Germany) based on the manufacturer’s guidelines. The concentration of isolated RNA was determined by spectrophotometric measurement at wavelengths of 260 nm (NanoDrop spectrophotometer, Thermo Scientific, MA, USA). The concentration of total RNA was assessed using the 260/280 nm absorption ratio; in all case the concentration was—as expected—at least 1.8. The quality and integrity of the RNA was verified in a Bioanalyzer 2100 (Agilent Technologies, Inc., Santa Clara, CA, USA). The RNA integrity numbers (RINs) ranged from 8.5 to 10, with a mean value of 9.2 (Agilent Technologies, Inc., Santa Clara, CA, USA). The RNA samples were diluted to a final concentration of 50 ng/μl. For the microarray study, 100 ng of good-quality, total RNA was used. The remaining RNA was exploited in the RT-qPCR validation.

### Microarray expression studies

The microarray experiment was conducted according to previously published procedures [19–22]. The protocol involving preparation of RNA for further hybridization was carried out using GeneChip WT PLUS Reagent Kit (Affymetrix, Santa Clara, CA, USA). The first stage included performing a two-step cDNA synthesis reaction from 100 ng RNA with the use of random primers extended by the T7 TNA polymerase promoter. The cRNA was synthesized during *in vitro* transcription (16h, 40°C) and then purified, re-transcribed into cDNA, and biotin labeled and fragmented using the Affymetrix GeneChip WT Terminal Labeling and Hybridization kit (Affymetrix, Santa Clara, CA, USA). Biotin-labeled fragments of cDNA were hybridized to the microarray probes included in the Affymetrix Human Gene 2.1 ST ArrayStrip (20h, 48°C). The microarrays were then washed and stained using the Affymetrix GeneAtlas Fluidics Station (Affymetrix, Santa Clara, CA, USA). The Imaging Station of GeneAtlas System (ThermoFisher Scientific, MA, USA) was used to scan the array strips. Preliminary analysis of the scanned chips was conducted with the Affymetrix GeneAtlas Operating Software (Affymetrix, Santa Clara, CA, USA). The software’s quality criteria were applied to verify the quality of the gene expression data.

### Microarray data analysis

The obtained CEL files were further analyzed using the R statistical language and Bioconductor package, including the relevant Bioconductor libraries. For the normalization, background correction, and calculation of the expression values of the examined genes, the Robust Multiarray Average (RMA) normalization algorithm implemented in the “Affy” library was applied [23]. A complete gene data table involving normalized gene expression values, gene symbols, gene names and Entrez IDs was generated on the basis of assigned biological annotations taken from the “pd.hugene.2.1.st” library. For the expression and statistical assessment, the linear models for microarray data included in the “limma” library was applied [24]. The established cut-off criteria were based on both differences in expression fold change (FC) greater than abs. 2 and an adjusted p value of ≤ 0.05. Genes fulfilling the aforementioned selection criteria were considered to be significantly different and therefore were subjected to the final analysis, described below in section 2.5.

Raw data files and a technical description were also deposited in the Gene Expression Omnibus (GEO) repository at the National Center for Biotechnology Information (http://www.ncbi.nlm.nih.gov/geo/) under the GEO accession number: GSE108035.

### Assignment of differentially expressed gene to relevant gene ontology (GO) terms

The set of significantly differentially expressed genes were subjected to functional annotation and clusterization using the Database for Annotation, Visualization and Integrated Discovery (DAVID) web-based bioinformatics tool, and they also underwent the Kyoto Encyclopaedia of Genes and Genomes (KEGG) pathway analysis [25]. Gene symbols describing differentially expressed genes were uploaded to DAVID through the “RDAVIDWebService” BioConductor library [26], where classification of significantly enriched ontological terms associated with the biological processes involved in regulating DNA damage and p53 signalling pathways was performed. The p values of specific GO terms were corrected using the Benjamini-Hochberg false discovery rate method and are reported as adj p-values. All results were visualized using the GOplot library [27]. Differentially expressed genes linked to specific GO terms were subjected to a hierarchical clusterization algorithm leading to their visualization as heatmaps. Finally, differentially expressed genes were also mapped to the signalling pathways of KEGG using the Pathview library, where expression folds of differentially expressed genes belonging to cell cycle and p53 signalling pathways were assigned to a predetermined colour scale in which upregulated and down-regulated genes are shown, respectively, in green and red color for the relevant experimental groups.

### RT-qPCR evaluation

Real Time-PCR reactions were performed using the PrimePCR^TM^ Custom Plates (Bio-Rad, CA, USA) and the specific synthesized primers for each gene: BTG2, RGCC, CDK2, CDKN2A and PMAIP1. cDNA samples were analyzed for genes of interest and for the reference gene glyceraldehyde 3-phosphate dehydrogenase (GAPDH). The expression level for each target gene was calculated as -2^ΔΔct^. The reaction was performed in triplicate for the gene of interest.

## Results

### Generation of chondrocyte-like cells from human induced pluripotent stem cells

**Fig 1:**
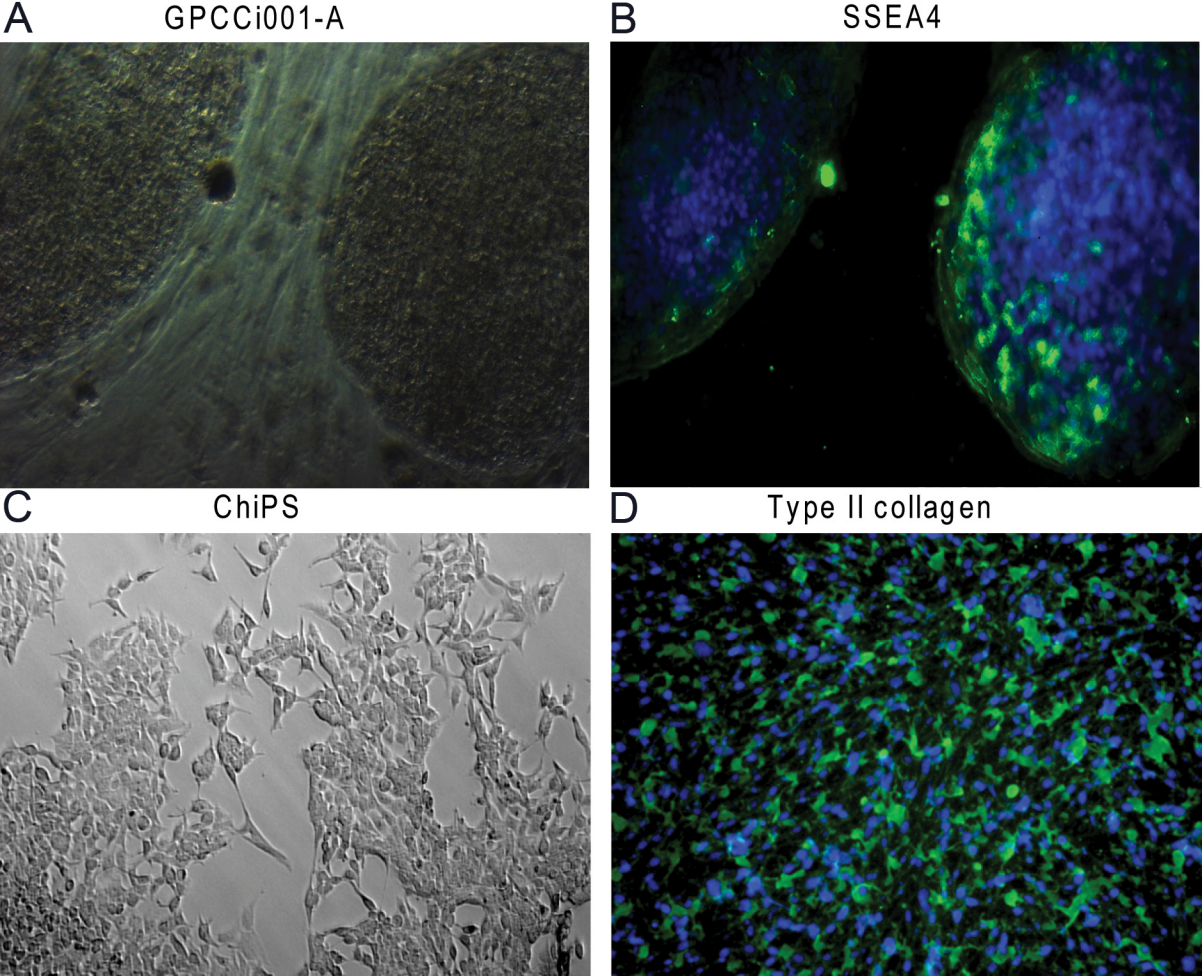
**GPCCi001-A cells (A) expressing pluripotency markers such as SSEA4 (B) were subjected to chondrogenic differentiation via monolayer culture with the set of defined growth factors.** The morphology of the obtained chondrocyte-like cells (ChiPS) presented characteristics of chondrocytes (C) and these cells contained chondrogenic markers such as type II collagen (D).

GPCCi001-A cells were generated from primary human dermal fibroblasts through a reprogramming process using the Stemcca-tetO lentiviral vector coding transcription factors responsible for pluripotency (Oct 4, Sox2, c-Myc and Klf4). GPCCi001-A cells express markers characteristic of pluripotent SCs and possess the capacity to form embryoid bodies with the derivatives of three primary germ layers [17] (Fig 1). HiPSCs cells were subjected to chondrogenic differentiation via monolayer culture based on a previously established protocol (DIRECT) [18]. Differentiated chondrocyte-like cells revealed features characteristic of chondrocytes such as type II collagen (Fig 1). To assess global gene expression, a microarray analysis using bioinformatics tools was performed. The global gene expression profile of ChiPS were compared to the hiPSCs (GPCCi001-A cell line) and to two cell lines of mature chondrocytes: HC-402-05a (Merck Millipore, Germany) (HC) and primary articular cartilage chondrocytes (ACC).

### Microarray gene expression profiling: Signalling pathways involving DNA damage and p53 are significantly upregulated in chondrocyte-like cells differentiated from hiPSCs

**Fig 2:**
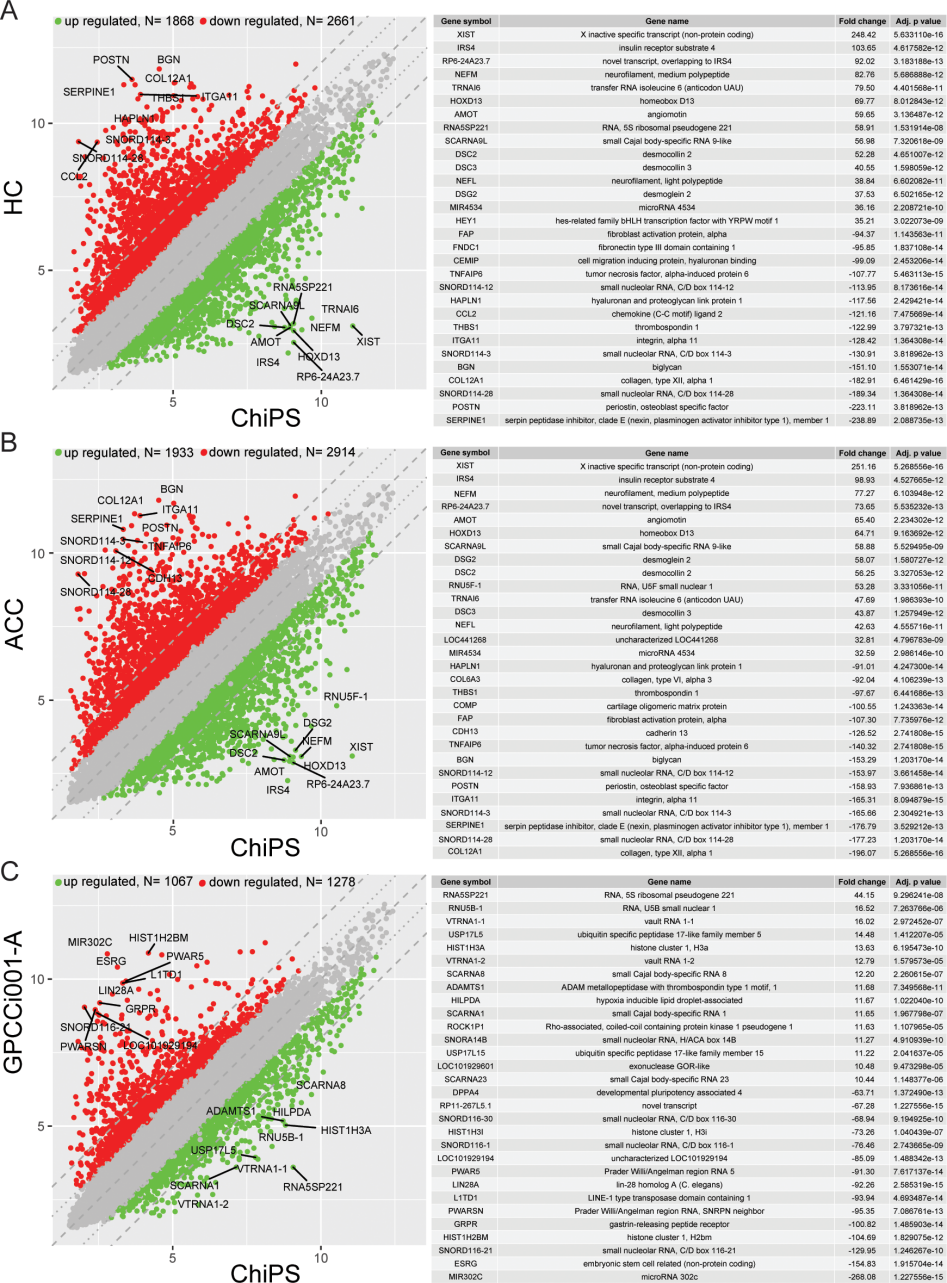
Gene expression profile based on microarray experiments. Variations in gene expression between the various comparisons—ChiPS vs HC-402-05a (HC) (A); ChiPS vs articular cartilage chondrocytes (ACC) (B); and ChiPS vs GPCCi001-A (C)—are shown as scatter plots. The x and y values on the scatter plots are the average normalized signal values, shown in a log_2_ scale. The red and green dots were set as fold change (FC) lines with a default change of 2.0. The tables show the 30 genes with the highest (15 genes) and lowest (15 genes) FC from the lists of differentially expressed genes (A,B,C).

Affymetrix Human Gene 2.1 ST Array Strips were used to analyse whole gene expression and to perform a complete comparison of ChiPS, HC, ACC, and GPCCi001-A transcriptome profiles (40,716 different transcripts). A pairwise scatter plot analysis was performed to determine the general profiles of the whole gene expression in the ChiPS, HC, ACC and GPCCi001-A experimental groups; this pairwise analysis revealed the patterns of upregulated and downregulated ChiPS-specific genes compared to the control HC, ACC and GPCCi001-A cells (Panel A, B, and C in Fig 2). Each colour dot corresponds to one differentially-expressed transcript, with red and green dots representing, respectively, the downregulated and upregulated genes in the ChiPS group. A gene expression FC > abs. 2 and an adjusted p value of ≤ 0.05 were considered to indicate significantly changed gene expression. Based on those criteria, the number of significantly changed genes in the ChiPS vs. HC, ACC and GPCCi001-A groups were, respectively, as follows: downregulated: 2661, 2914, 1278 and upregulated: 1868, 1933, 1067. The genes with the highest and lowest FC values (the 15 most upregulated and 15 most downregulated genes per comparison) from all of the groups (ChiPS vs HC, ACC and GPCCi001-A) are presented in tabular formats with the gene symbol, gene name, FC, and adjusted p value (Panel A, B, and C in Fig 2) The FC values for these genes were extremely high: for the ChiPS vs HC groups, the FC values of upregulated genes ranged from 35.21 to 248.42 while the range for downregulated genes was from -238.89 to -94.37. The genes that demonstrated the greatest upregulation (p<0.05) were: RNA expression of X inactive specific transcript (non-protein coding) (248.42); insulin receptor substrate 4 (103.65); and novel transcript overlapping to IRS4 (92.02). The genes with the greatest downregulation were: serpin peptidase inhibitor, clade E (nexin, plasminogen activator inhibitor type 1), member 1 (-238.89); periostin, osteoblast specific factor (-223.11); small nucleolar RNA, C/D box 114–28 (-189.34) (p<0.05). In the ChiPS vs ACC groups, the values of the most upregulated genes ranged from 32.59 to 251.16 and from -196.07 to -91.01 for downregulated genes. The X inactive specific transcript (non-protein coding) (251.16), insulin receptor substrate 4 (98.93), and neurofilament, medium polypeptide (77.27) genes showed the greatest increase in gene expression level (p<0.05). By contrast, the most downregulated genes (p<0.05) were collagen, type XII, alpha 1 (-196.07); small nucleolar RNA, C/D box 114–28 (-177.23); and serpin peptidase inhibitor, clade E (nexin, plasminogen activator inhibitor type 1), member 1 (-176.79). In the comparison between the ChiPS and GPCCi001-A groups, the most notable increases were in the RNA expression of RNA 5S ribosomal pseudogene 221 (44.15); RNA, U5B small nuclear 1 (16.52) and the vault RNA 1–1 genes (16.02) (p<0.05). By contrast, the most downregulated genes were microRNA302c, embryonic stem cell related (non-protein coding), and small nucleolar RNA, C/D box 116–21, with respective FC values of -268.08, -154.83, and -129.95 (p<0.05).

**Fig 3:**
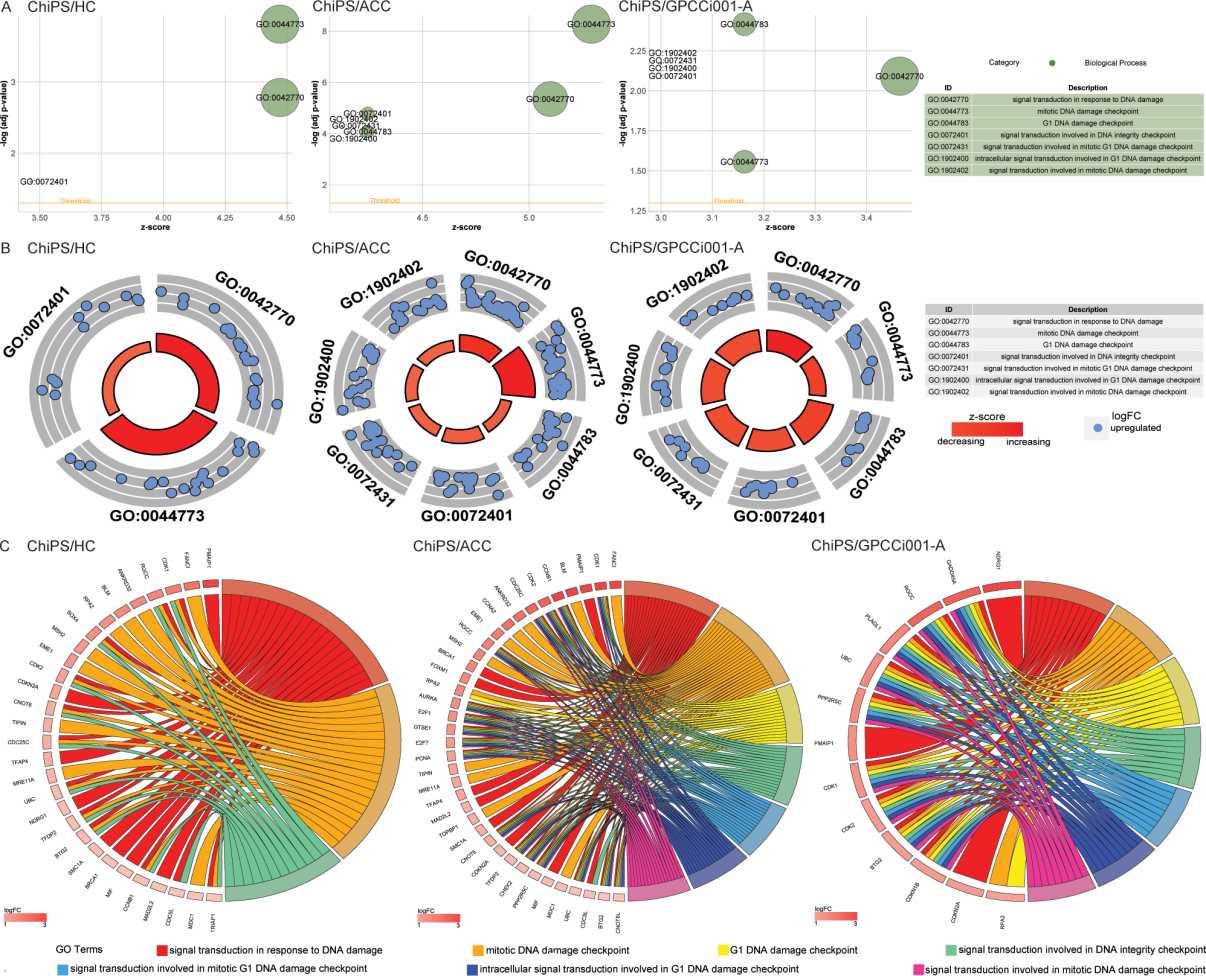
Bubble plots of significantly-enriched GO terms for the experimental groups: ChiPS vs HC-402-05a (HC), articular cartilage chondrocytes (ACC), and GPCCi001-A related to DNA damage repair (DDR) mechanisms activated during chondrogenic differentiation. These activated mechanisms were as follows: “signal transduction in response to DNA damage”; “mitotic DNA damage checkpoint”; “G1 DNA damage checkpoint”; “signal transduction involved in DNA integrity checkpoint”; “signal transduction involved in mitotic G1 DNA damage checkpoint”; “intracellular signal transduction involved in G1 DNA damage checkpoint”; and “signal transduction involved in DNA damage checkpoint”. The negative logarithm of the adjusted p-values from all analysed GO terms are shown on the y-axes, while the z-score values are given on the x-axes (A). The circular scatter plots show the differentially expressed genes involved in specific GO terms. The logarithm of the fold change value (logFC) of differentially expressed genes is shown (B). Circos plots show the interdependence between selected GO terms and their genes. The genes are shown on the left side of the graph and indicated by their symbols and ordered by logFC values (C).

**Fig 4:**
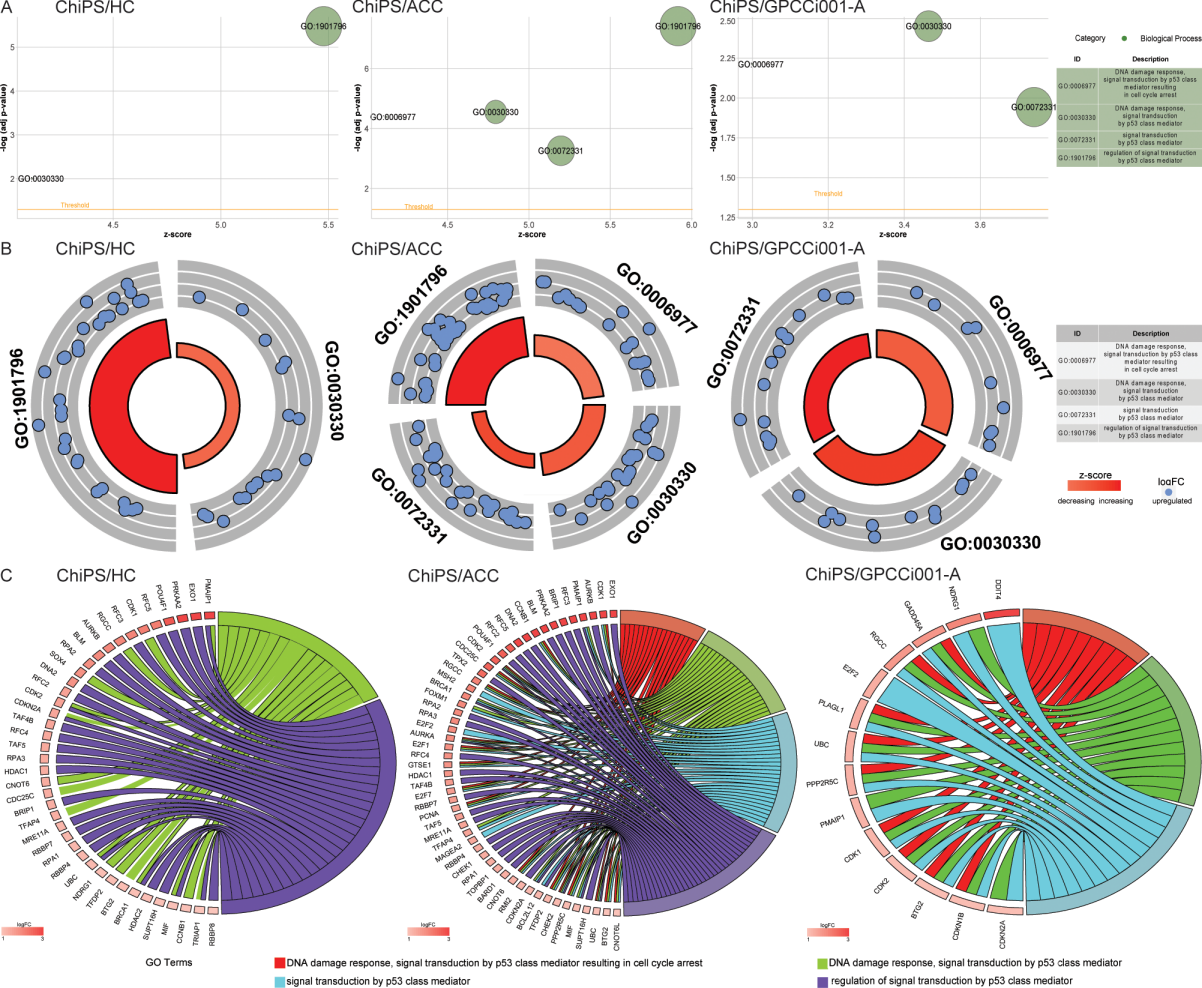
Bubble plots of significantly-enriched GO terms for the experimental groups: ChiPS vs HC-402-05a (HC), articular cartilage chondrocytes (ACC), and GPCCi001-A related to DNA damage repair (DDR) mechanisms activated during chondrogenic differentiation. These activated mechanisms were as follows “DDR signal transduction by p53 class mediator resulting in cell cycle arrest”; “DDR signal transduction by p53 class mediator”; “signal transduction by p53 class mediator” and “regulation of signal transduction by p53 class mediator”. The negative logarithm of the adjusted p-values from all analysed GO terms are shown on the y-axes, while the z-score values are given on the x-axes (A). The circular scatter plots show the differentially expressed genes involved in specific GO terms. The logarithm of the fold change value (logFC) of differentially expressed genes is shown (B). Circos plots show the interdependence between selected GO terms and their genes. Genes are situated on the left side of the graph and indicated by their symbols and ordered by logFC values (C).

Differentially expressed gene sets from the comparisons (ChiPS vs HC, ACC, GPCCi001-A cells) were assigned to significantly enriched GO terms associated with biological processes engaged in DNA damage response (DDR), as follows: “signal transduction in response to DNA damage”; “mitotic DNA damage checkpoint”; “G1 DNA damage checkpoint”; “signal transduction involved in DNA integrity checkpoint”; “signal transduction involved in mitotic G1 DNA damage checkpoint“; “intracellular signal transduction involved in G1 DNA damage checkpoint”; “signal transduction involved in mitotic DNA damage checkpoint”; “DDR to signal transduction by p53 class mediator resulting in cell cycle arrest”; “DDR signal transduction by p53 class mediator”; “signal transduction by p53 class mediator”; and “regulation of signal transduction by p53 class mediator” (Table 1). The GOplot library was used to visualize these results [27]. The general characteristics of enriched GO term are presented as bubble plots with the negative logarithms of the adj p-values on the y-axes and Z-score values on the x-axes. The Z-score, which indicates stimulated (positive value) or inhibited (negative value) processes, was calculated automatically using the GOplot library. In the ChiPS group, gene sets assigned to specific ontological groups demonstrated a notable increase in gene expression, as reflected by positive Z-score values (Panel A in Figs 3 and 4)

**Table 1:** Significantly differentiated genes from all experimental groups assigned to specific GO terms involved in DNA damage and p53 signalling pathways.

Biological process	Number of differentially expressed genes and adj p-value		
	ChiPS vs HC	ChiPS vs ACC	ChiPS vs GPCCi001-A
**Signal transduction in response to DNA damage**	20 (adj p = 0.002)	26 (adj p = 4.43E-06)	12 (adj p = 0.008)
**Mitotic DNA damage checkpoint**	20 (adj p = 0.0001)	28 (adj p = 5.33E-09)	10 (adj p = 0.027)
**G1 DNA damage checkpoint**	-	18 (adj p = 8.28E-05)	10 (adj p = 0.004)
**Signal transduction involved in DNA integrity checkpoint**	12 (adj p = 0.02)	18 (adj p = 1.63E-05)	9 (adj p = 0.008)
**Signal transduction involved in mitotic G1 DNA damage checkpoint**	-	17 (adj p = 5.08E-05)	9 (adj p = 0.007)
**Intracellular signal transduction involved in G1 DNA damage checkpoint**	-	17 (adj p = 5.08E-05)	9 (adj p = 0.007)
**Signal transduction involved in mitotic DNA damage checkpoint**	-	17 (adj p = 5.08E-05)	9 (adj p = .0.007)
**DNA damage response, signal transduction by p53 class mediator resulting in cell cycle arrest**	-	17 (adj p = 4.08E-05)	9 (adj p = 0.006)
**DNA damage response, signal transduction by p53 class mediator**	17 (adj p = 0.01)	23 (adj p = 2.78E-05)	12 (adj p = 0.003)
**Signal transduction by p53 class mediator**	-	27 (adj p = 0.0005)	14 (adj p = 0.01)
**Regulation of signal transduction by p53 class mediator**	30 (adj p = 3.32E-06)	35 (adj p = 3.82E-08)	-

The logarithms of the FC values for the differentially expressed genes corresponding to GO terms are shown in Panel B in Fig 4. Each blue circle presents an upregulated gene from the specific GO terms. In many cases, single genes are often assigned to many ontological terms and therefore the complex relationships between genes and GO terms were mapped using circos plots including logFC values and gene symbols (Panel C in Figs 3 and 4).

**Fig 5:**
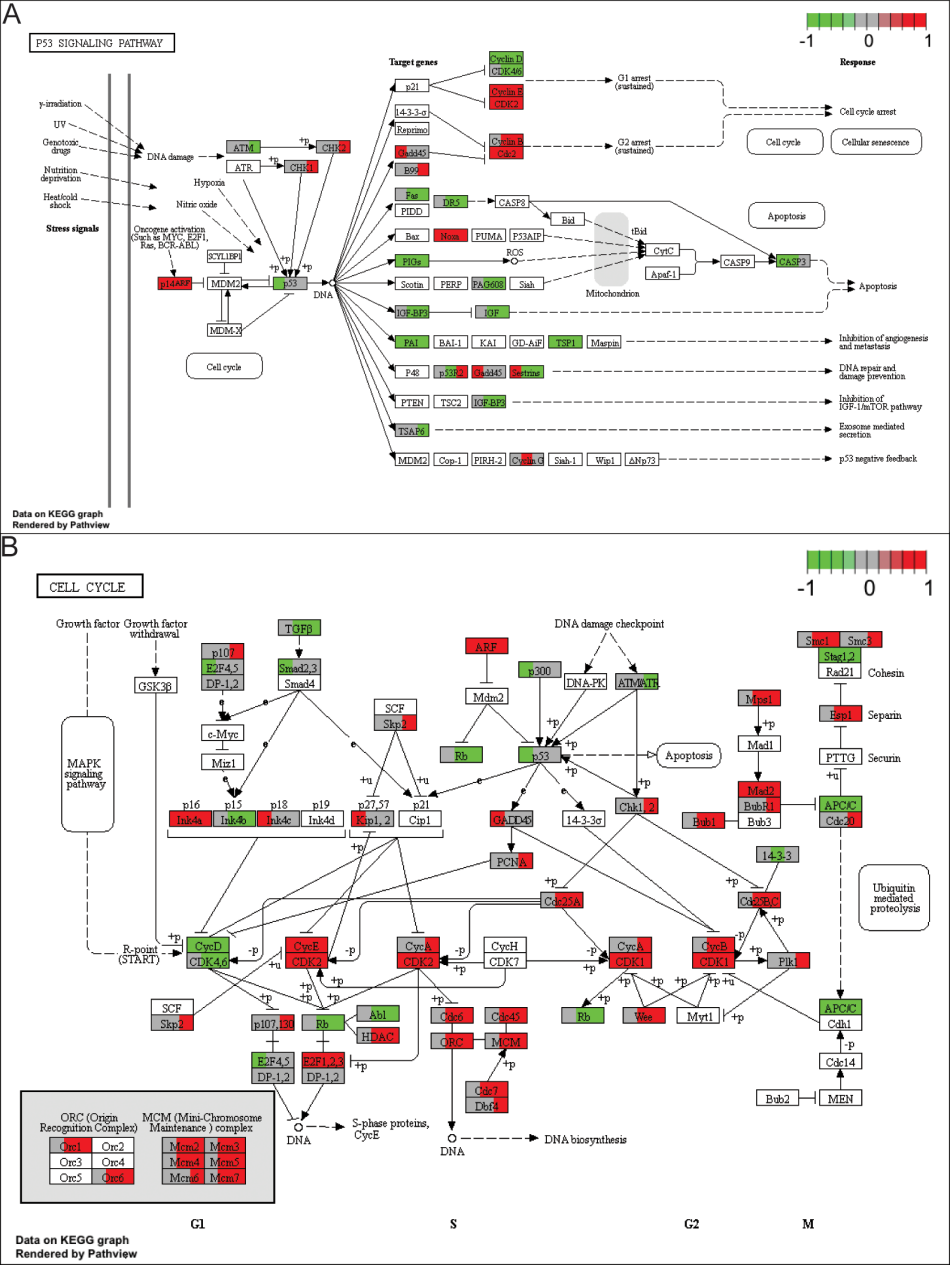
Kyoto Encyclopedia Genes and Genomes (KEGG) pathway analysis of differentially expressed genes. The significant pathways: “p53” (A) and “cell cycle” (B) of regulated genes are demonstrated. Differentially expressed genes belonging to these pathways were assigned to a predetermined colour scale, which was subsequently imposed on the gene/protein symbol field (left third for ChiPS vs HC, middle third for ChiPS vs ACC, right third for ChiPS vs GPCCi001-A). The green and red colors indicate, respectively, upregulated and downregulated gene expression in the relevant comparisons.

A pathway analysis was also performed for the differentially expressed genes based on the KEGG database. This analysis allowed us to determine the biological pathways “p53” and “cell cycle”, which involve a significant enrichment of differentially expressed genes in the ChiPS group (p<0.05). Differentially expressed genes belonging to these pathways were assigned to a predetermined colour scale, which was subsequently imposed on the gene/protein symbol field (Fig 5).

**Fig 6:**
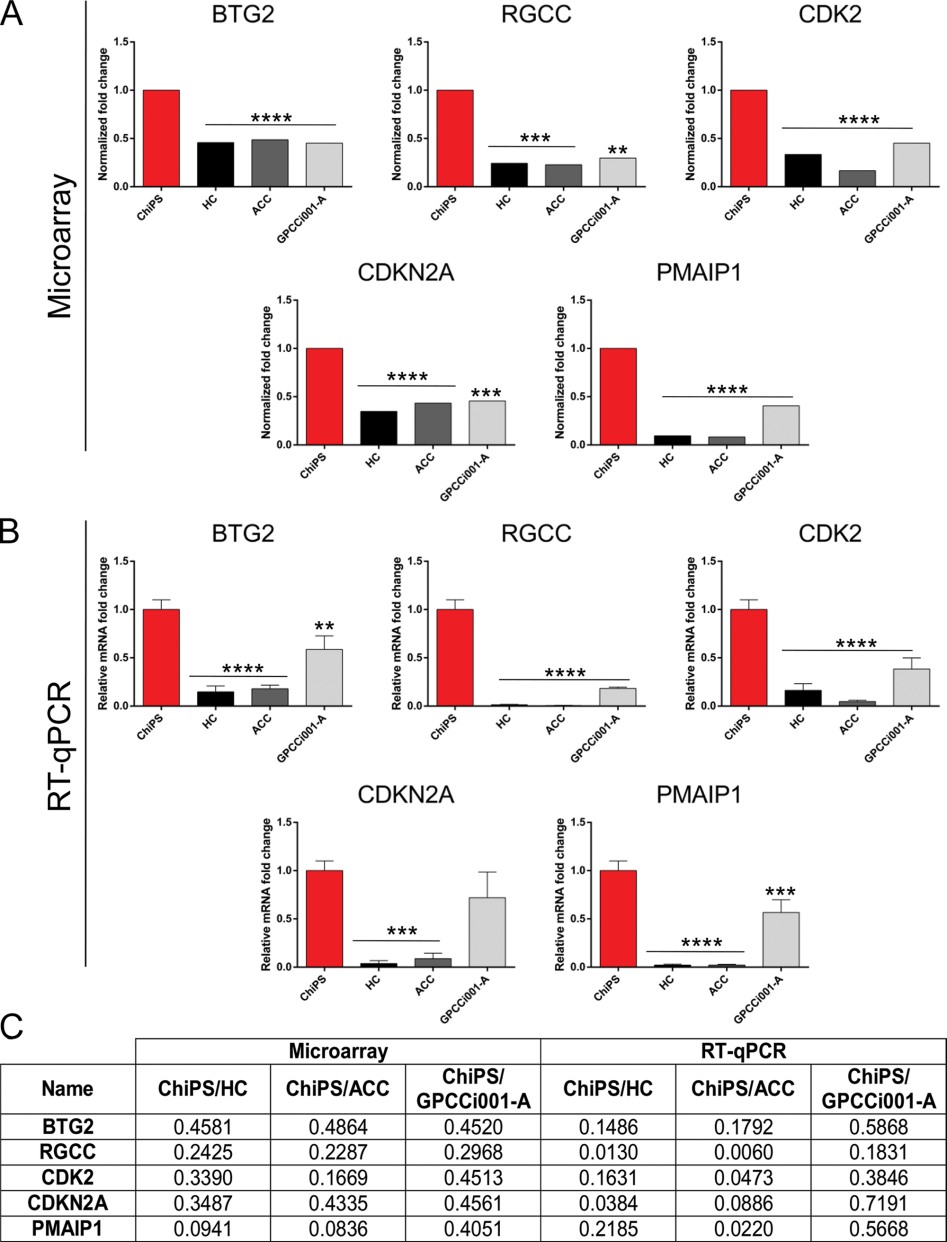
Real time qPCR validation of microarray data. For the evaluation, we selected the most highly expressed genes involved in both DNA damage and p53 signalling pathways based on the previously created circos plot GO terms. The top panel represents normalized ChiPS fold changes (FC) of selected genes based on microarray data (A). The bottom panel represents microarray data assessed by RT-qPCR technique. The graph represents means ± SD from three replicates per group (B). The table shows normalized FC and p-values of selected genes from the microarray and RT-qPCR analyses (C).

Based on the circos plots that revealed the most upregulated genes in the ChiPS vs HC, ACC and GPCCi001-A groups, we selected the most upregulated genes (BTG2, RGCC, CDK2, CDKN2A and PMAIP1) for further RT-qPCR evaluation. All of these genes are involved in DDR signalling pathways (Panels A, B, and C in Fig 6). Expression of genes engaged in activating p53 and DNA damage signalling pathways was significantly higher in the ChiPS compared to the control groups (HC, ACC and GPCCi001-A cells).

Significantly differentially expressed genes from each GO term were also subjected to a hierarchical clusterization algorithm and visualized as heatmaps. Arbitrary signal intensities from the most up- or down-regulated genes are presented by colours (green = higher expression, red = lower expression) and described by their symbols. Log2 signal intensity values for any single gene were resized to the row Z-Score scale (Panels A, B, and C in S1 and S2 Figs).

## Discussion

Our group previously established a novel protocol to obtain chondrocyte-like cells from hiPSCs derived from primary human dermal fibroblasts via a reprogramming process [17,18]. In the current study, analysed the global gene expression of ChiPS, HC, ACC and GPCCi001-A cells, with a focus on the stress-induced signalling pathways activated during the chondrogenic process *in vitro*. The main aim of this study was to assess how the differentiation process influences key genes engaged in DNA damage and p53 signalling pathways, comparing the results obtained for the ChiPS to those obtained for adult chondrocytes and hiPSCs. Our findings show that chondrogenic differentiation *in vitro* provokes an important stress-induced response for these cells, leading to DNA breaks and activation of major components of the p53 signalling pathway. Published data on chondrogenic differentiation of hiPSCs and the effect of this process on DDR mechanisms are scant. For this reason, we discuss our findings in the context of the available data regarding the regulation of p53 and DDR processes, most of which are from mouse models and human SCs in a range of distinct differentiation processes.

Lin et al. reported that rapid downregulation of Nanog—one of the key pluripotent transcription factors—during the differentiation of mouse embryonic stem cells (ESCs) correlates with the induction and Ser 315 phosphorylation of p53. The increased activity of p53 helps to maintain the genetic stability of mouse ESCs, which—as a result of the differentiation process *in vitro—*can give rise to other cell types. ESC-derived mouse cells, in contrast to undifferentiated mouse ESCs, have the capacity to undergo p53-dependent cell-cycle arrest and apoptosis [28].

According to Armesillia-Diaz et al. (2009), the p53 signalling pathway plays an important role in regulating the differentiation process in mouse bone marrow-derived mesenchymal stem cells (MSCs). In that study, murine MSCs with p53 knockout presented genomic instability involving increased expression of c-Myc and the time required by these cells to differentiate into adipocytes and osteocytes was significantly reduced [29].

P53 interacts with key regulators of neurogenesis to redirect mouse neural SCs (NSCs) to differentiate as an alternative to cell death during stress. When this occurs, the histone H3 lysine 27-specific demethylase JMJD3 induces p53 stabilization and nuclear accumulation through ARF-dependent mechanisms [30]. The available evidence [31] indicates that p53—a key factor in the regulation of neuronal differentiation of NSCs in organotypic cultures of mouse hippocampus and the p53 signalling pathway—are probably regulated by proteins associated with cell cycle regulation (e.g., Pim-1 and Phb1) [31]. Another group [32] reported that p53 is strongly involved in embryonic development by mediating the p38 mitogen-activated protein kinase (p38 MAPK) pathway during early differentiation of mouse ESCs into mesodermal and neural lineages. The p53 has the capacity to suppress expression of Nanog [32].

In the nucleus of hESCS, p53 expression is scarcely detectable due to HDM2 and TRIM24 negative regulators, which leads to p53 degradation. After differentiation has been induced, lysine 373 of p53 is acetylated by CBP/p300, thus disrupting the interaction between p53 and negative regulators, which ultimately stabilizes and activates p53. Activated p53 induces expression of p21—the cell cycle regulator—which is responsible for the accumulation of differentiated hESCs in the gap (G1) phase of the cell cycle. In addition, p53 promotes miR-34a and miR-145, which inhibit expression of the SC factors Oct4, Klf4, Lin28A and Sox2 in differentiated hESCs [33].

A comprehensive study involving the role of p53 in early hESC differentiation was carried out by Akdemir and collaborators [34]. Those authors performed genome-wide profiling of protein interactions with chromatin and the intersection with global gene expression analysis to investigate potential networks of transcription and epigenetic regulators of pluripotency and self-renewal of hESCs. Based on the genome-wide profiling, they showed that differentiation-activated p53 targets are bound by core pluripotency factors—that is, Oct4 and Nanog at chromatin enriched in H3K27me3 and H3K4me3. The conclusion of that study was that p53 mediates the expression of specific developmental genes [34].

Both p53 and p73 have been shown to play an important role in regulating apoptosis in mouse ESCs in response to DNA damage or differentiation. Importantly, neither p53 nor its target p73 is required for G2/M arrest. This indicates that the major role of p53 in maintaining genetic stability of mouse ESCs is achieved by eliminating damaged cells [35]. In our study, we observed a notable regulation of genes engaged in the p53 signalling pathway and the cell cycle in ChiPS, in which cell cycle phases (such as mitotic and G1 DNA damage checkpoints) were activated, with cell cycle arrest in response to chondrogenic differentiation *in vitro*.

The p53, which has been described as “a guardian of the genome”, is a key factor responsible for the safety of adult cells because it induces cell-cycle arrest and apoptosis during stress. Moreover, p53 plays an important role in pluripotent SC differentiation and in reprogramming, leading to the generation of hiPSCs. DNA damage strongly activates the p53 signalling pathway, thereby inhibiting pluripotent transcription factors while promoting differentiation. Pluripotent SCs are capable of transforming into malignant teratomas, carcinomas or cancer SCs when p53 is not controlled. On the other hand, p53 has the ability to effective inhibit cancer by suppressing pluripotent SC-mechanisms [36,37]. For these reasons, it is essential to carefully assess p53-related activities in pluripotent SC-based research and applications. Although the aforementioned studies concerning activation of p53 are highly informative, they do not, unfortunately, provide unambiguous data that would allow us to reach definitive conclusions about stress-induced DDR mechanisms in SCs undergoing differentiation process *in vitro*.

Saretzki et al. (2008) provided valuable data on the repression of stress defence mechanisms during spontaneous differentiation of hESCs. Those authors discovered that the differentiation process *in vitro* leads to a notable downregulation of telomerase activity due to deacetylation of histones H3 and H4 at the hTERT promoter as well as deacetylation of histone H3 at the hTR promoter. Those authors also reported a higher level of mitochondrial superoxide production and cellular levels of ROS during differentiation. Nevertheless, in that study they did not observe an increased expression of major antioxidant genes. They did, however, find evidence indicating an accumulation of DNA damage in hESCs undergoing differentiation *in vitro* and a significant decrease in the expression of genes involved in the main DNA repair mechanisms. Those findings suggest that hESC-derived cells are less proficient than hESCs in protecting their genome [15], an observation that is partially confirmed by our results, particularly the strong correlation between DNA damage and p53 activation in hiPSCs during chondrogenic differentiation *in vitro*.

## Conclusions

This study evaluated chondrocyte-like cells (ChiPS) derived from the GPCCi001-A cell line via a chondrogenic process. We found that the chondrogenic process *in vitro* is controlled by p53, which is mainly responsible for diminishing pluripotency factors and for preventing hiPSC-derived cells to backslide into the pluripotency state. Our findings also demonstrate that hiPSCs undergoing chondrogenic differentiation present significantly higher levels of DNA damage, which may be ascribed to differentiation-associated stress. We found that numerous genes engaged in cell cycle arrest and checkpoint were significantly up-regulated in the ChiPS as a response to accumulated DNA damage and activation of the p53 signalling pathway.

Although the precise nature of the cells obtained through chondrogenic differentiation *in vitro* of hiPSCs remains an open question, the ChiPS assessed in this study present features that are characteristic of cells involved in chondrogenesis. However, the use of such cells in regenerative medicine could present a risk given that ChiPS may be less proficient in maintaining their genetic stability; this characteristic, if confirmed, may constitute a serious drawback for the future use of these cells in clinical practice.

## Supporting information

S1 Fig **Heatmap graphs of the genes in the experimental groups: ChiPS vs HC-402-05a (HC) (A), articular cartilage chondrocytes (ACC) (B), and GPCCi001-A (C) from the specific GO terms**. The significant GO terms were as follows: “signal transduction in response to DNA damage”; “mitotic DNA damage checkpoint”; “G1 DNA damage checkpoint”; “signal transduction involved in DNA integrity checkpoint”; “signal transduction involved in mitotic G1 DNA damage checkpoint”; “intracellular signal transduction involved in G1 DNA damage checkpoint”; and “signal transduction involved in DNA damage checkpoint”. Arbitrary signal intensity obtained from the microarray analysis is represented by the appropriate colours (green = higher expression; red = lower expression). Log2 signal intensity values for each gene were resized to row Z-score scales. Genes belonging to the relevant GO term are described by their symbols (A,B,C).
(TIF)

S2 Fig **Heatmap graphs of the genes in the experimental groups: ChiPS vs HC-402-05a (HC) (A), articular cartilage chondrocytes (ACC) (B), and GPCCi001-A (C) from the specific GO terms.** The significant GO terms were as follows: “DNA damage response signal transduction by p53 class mediator resulting in cell cycle arrest”; “DNA damage response signal transduction by p53 class mediator”; “signal transduction by p53 class mediator”; and “regulation of signal transduction by p53 class mediator”. Arbitrary signal intensity obtained from the microarray analysis is represented by the appropriate colours (green = higher expression; red = lower expression). Log2 signal intensity values for each gene were resized to row Z-score scales. Genes belonging to the relevant GO term are described by their symbols (A,B,C).
(TIF)
