# Supplementary figures and images for: Forced differentiation in vitro leads to stress-induced activation of DNA damage response in hiPSC-derived chondrocyte-like cells
Source: PLoS One. 2018 Jun 4;13(6):e0198079. doi: 10.1371/journal.pone.0198079 (PMC5986142; doi:10.1371/journal.pone.0198079)

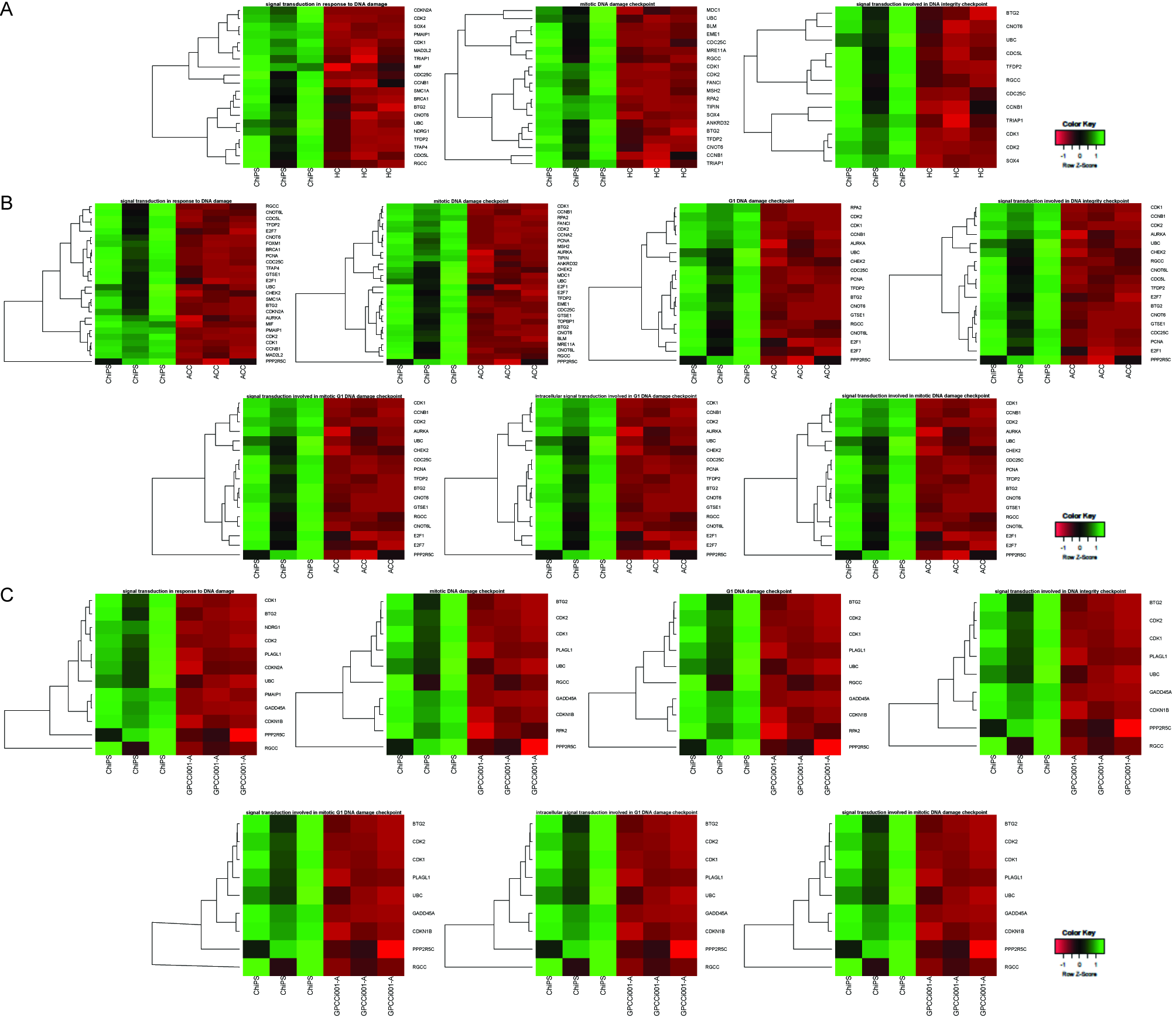

Supplement: S1 Fig — Heatmap graphs of the genes in the experimental groups: ChiPS vs HC-402-05a (HC) (A), articular cartilage chondrocytes (ACC) (B), and GPCCi001-A (C) from the specific GO terms. The significant GO terms were as follows: “signal transduction in response to DNA damage”; “mitotic DNA damage checkpoint”; “G1 DNA damage checkpoint”; “signal transduction involved in DNA integrity checkpoint”; “signal transduction involved in mitotic G1 DNA damage checkpoint”; “intracellular signal transduction involved in G1 DNA damage checkpoint”; and “signal transduction involved in DNA damage checkpoint”. Arbitrary signal intensity obtained from the microarray analysis is represented by the appropriate colours (green = higher expression; red = lower expression). Log2 signal intensity values for each gene were resized to row Z-score scales. Genes belonging to the relevant GO term are described by their symbols (A,B,C). (TIF) [file pone.0198079.s001.tif]

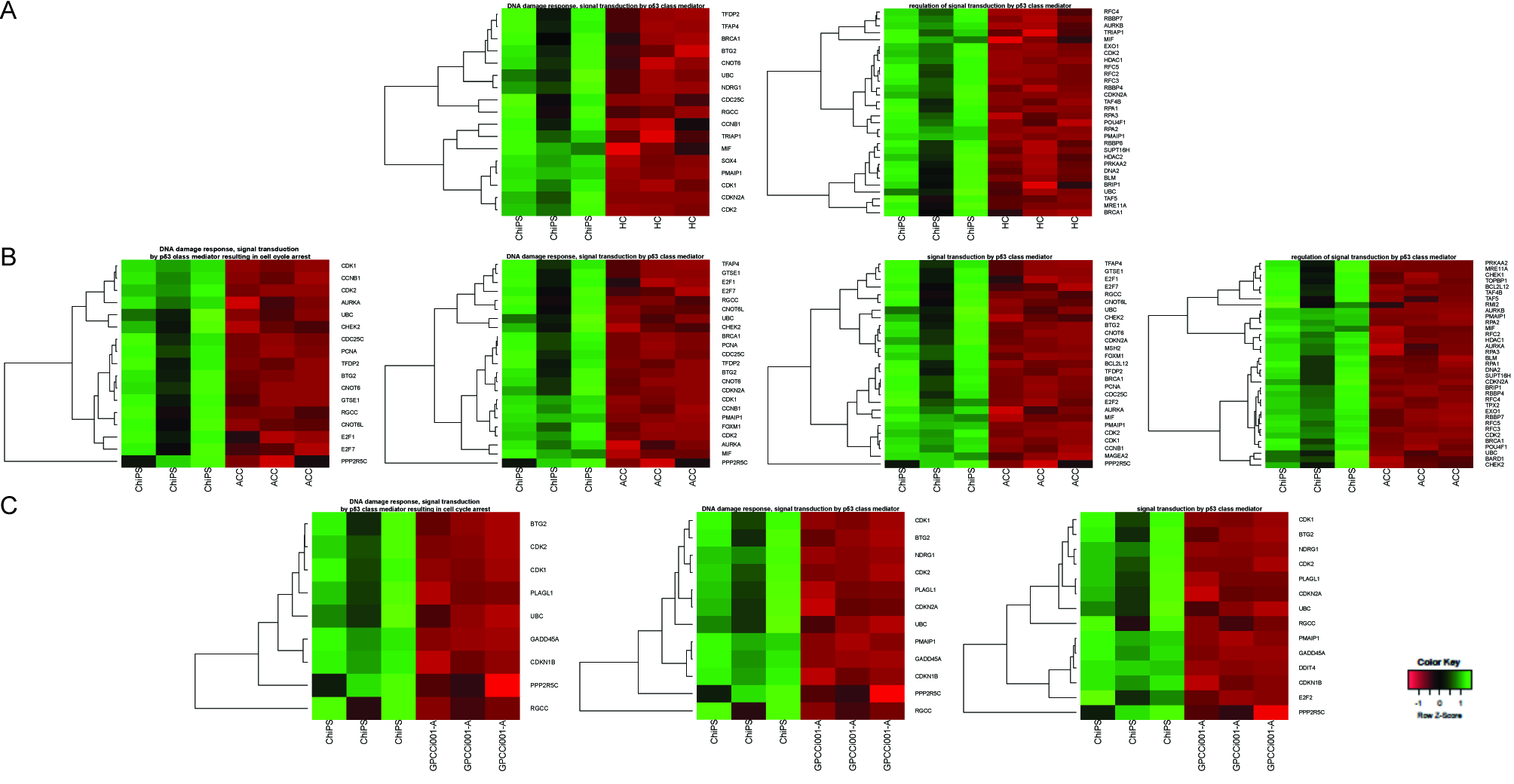

Supplement: S2 Fig — Heatmap graphs of the genes in the experimental groups: ChiPS vs HC-402-05a (HC) (A), articular cartilage chondrocytes (ACC) (B), and GPCCi001-A (C) from the specific GO terms. The significant GO terms were as follows: “DNA damage response signal transduction by p53 class mediator resulting in cell cycle arrest”; “DNA damage response signal transduction by p53 class mediator”; “signal transduction by p53 class mediator”; and “regulation of signal transduction by p53 class mediator”. Arbitrary signal intensity obtained from the microarray analysis is represented by the appropriate colours (green = higher expression; red = lower expression). Log2 signal intensity values for each gene were resized to row Z-score scales. Genes belonging to the relevant GO term are described by their symbols (A,B,C). (TIF) [file pone.0198079.s002.tif]
